# Encoding and decoding spatio-temporal information for super-resolution microscopy

**DOI:** 10.1038/ncomms7701

**Published:** 2015-04-02

**Authors:** Luca Lanzanò, Iván Coto Hernández, Marco Castello, Enrico Gratton, Alberto Diaspro, Giuseppe Vicidomini

**Affiliations:** 1 Nanoscopy, Nanophysics Istituto Italiano di Tecnologia, via Morego 30, Genoa 16163, Italy; 2 Department of Physics, University of Genoa, via Dodecaneso 33, Genoa 16146, Italy; 3 Department of Computer Science, Bioengineering, Robotics and Systems Engineering, via Opera Pia 13, Genoa 16145, Italy; 4 Laboratory for Fluorescence Dynamics, Department of Biomedical Engineering, University of California, Irvine, California 92697, USA

## Abstract

The challenge of increasing the spatial resolution of an optical microscope beyond the diffraction limit can be reduced to a spectroscopy task by proper manipulation of the molecular states. The nanoscale spatial distribution of the molecules inside the detection volume of a scanning microscope can be encoded within the fluorescence dynamics and decoded by resolving the signal into its dynamics components. Here we present a robust and general method to decode this information using phasor analysis. As an example of the application of this method, we optically generate spatially controlled gradients in the fluorescence lifetime by stimulated emission. Spatial resolution can be increased indefinitely by increasing the number of resolved dynamics components up to a maximum determined by the amount of noise. We demonstrate that the proposed method provides nanoscale imaging of subcellular structures, opening new routes in super-resolution microscopy based on the encoding/decoding of spatial information through manipulation of molecular dynamics.

## Abstract (web summary)

Increasing the resolution of fluorescence microscopy is a fundamental need for modern cell biology. Lanzanò *et al.* demonstrate that arbitrary spatial resolution is, in principle, possible by encoding the fluorophore's spatial distribution information in the temporal dynamics of the fluorophore's transition.

## Graphical abstract

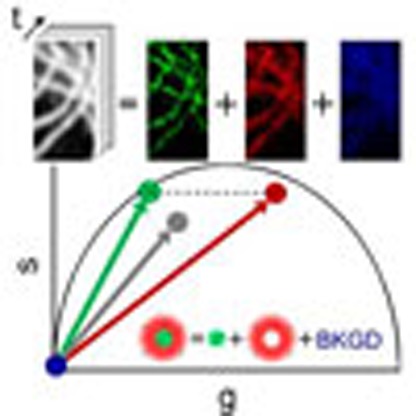

The visualization of macromolecular complexes inside cells is a fundamental need in modern cell biology. The fact that the size of interest is in the range of 10 to 100 nm generated a growing interest for fluorescence microscopy methods able to form images with spatial resolution well below the diffraction limit (~200 nm). Since the early 1990s, several physical concepts were identified for breaking the diffraction limit, which triggered the development of super-resolved fluorescence microscopy^1^,^2^,^3^. The spatial resolution of a microscope has usually been expressed in terms of its point-spread-function (PSF) (the image of a point-like source), stating that fluorophores that are closer than the PSF size cannot be resolved. With few exceptions (for instance, methods based on non-classicality of light^4^ and near-field interaction^5^) all the super-resolution fluorescence methods provide subdiffraction resolution by transiently switching the fluorophores between optically distinguishable states and causing the fluorophores within the same diffraction-limited- (DL-) PSF-sized region to signal sequentially. Accordingly, the different super-resolution concepts can be broadly classified based on how the fluorophore states are manipulated^6^. In methods that employ stochastic switching and readout, where the DL-PSF represents the image of a fluorophore, only one fluorophore, at that time, is likely to fluoresce in a region covered by the DL-PSF^7^,^8^,^9^, so that its position can be determined with a precision much higher than the DL-PSF size^10^. In methods that employ targeted switching and readout, where the DL-PSF represents the focal volume of the scanned excitation beam, the fluorophores are selectively silenced at the periphery of the DL-PSF, resulting in an effective- (E-) PSF of smaller size^11^,^12^. Notably, structured illumination super-resolution techniques can be classified as targeted methods^13^,^14^,^15^, since they can be regarded as a parallelization of the single-point scanning methods.

The need for a generic super-resolution technique to preclude the simultaneous emission of neighbouring fluorophores comes from the fact that the fluorescence signal versus the spatial coordinate is the most prominent physical quantity used for the construction of an image. Since at each pixel a given number of photons *N* are detected originating from an undetermined number of fluorophores at unknown positions within the DL-PSF-sized region, one way to reduce this indetermination is to act on the fluorophores either by reducing their number to one (stochastic switching) or by restricting their possible positions within the DL-PSF-sized region (targeted switching). However, adding a temporal dimension to the measure of fluorescence can make this concept based on restricting the number/position of fluorophores superfluous^16^,^17^,^18^. This can be addressed in terms of information capacity theory^19^: subdiffraction resolution can be obtained with a method that encodes information from the saturated spatial channels of the microscope system into the temporal channel and decodes it after the transmission^20^,^21^.

To the best of our knowledge, the first super-resolution approach making explicit use of the temporal dynamics of fluorescence was proposed by Enderlein (ref. 16). This approach, called dynamic saturation optical microscopy (DSOM), relies on the fact that, on modulated illumination and in the presence of dark-state fluorophore transitions, a spatial distribution of the illumination intensity induces a spatial-dependent temporal dynamics. A confocal-based implementation has been realized exploiting the singlet–triplet-state transition^22^. Unfortunately, an efficient DSOM implementation needs hundreds of microseconds triplet-state lifetime fluorophores, which favours the occurrence of unwanted photo-bleaching processes.

A similar spatial-dependent temporal dynamics can be obtained exploring the singlet states transitions and using the process of stimulated emission (SE). The (singlet) excited-state lifetime *τ* of a fluorophore reduces as a function of the intensity of the light beam inducing the SE process, the so-called stimulated emission depletion (STED) beam. Hence the spatial distribution of the intensity of the STED beam, will determine differences in the fluorescence decay rates of fluorophores at different positions within the DL-PSF. This principle has been used recently to implement an efficient and versatile STED microscope: the gated continuous-wave-STED (CW-STED) microscope^23^,^24^,^25^. In a STED microscope, the STED beam is shaped like a doughnut and co-aligned with the regular Gaussian excitation beam. If the STED beam operates in CW (the CW-STED implementation) the intensity is normally too low to effectively quench all the fluorophores in the periphery and thus to significantly reduce the size of the E-PSF. However, the STED beam modifies the fluorescence lifetime at the periphery but not at the centre of the E-PSF, so that by using a pulsed excitation beam and a time-resolved measurement the collection of fluorescent photons after a time-delay *T*_g_ from the excitation events (time-gated detection) allows the removal of a significant fraction of photons emitted in the periphery. Theoretically, the spatial resolution of the gated CW-STED microscope improves with the time-delay *T*_g_, but since ‘wanted’ photons, stemming from the E-PSF centre, are also discarded the limiting factor becomes the signal-to-noise ratio (SNR)^26^. This can be made worse by the presence of uncorrelated background signal, originating for instance from direct excitation by the STED beam, which is not removed by simple time gating^27^,^28^,^29^. The very same principle of gated CW-STED is the basis of another super-resolution configuration, which uses a regular Gaussian STED beam^30^ . In this implementation, the time windows of the time-resolved measurements correspond to images recorded with their own E-PSFs, which form a basis set from which an optimized E-PSF is obtained and can be used for reconstructing an image with subdiffraction spatial resolution^30^. Notwithstanding this, despite the relatively simple optical set-up implementation, the optimization of the parameters of the reconstructed E-PSF for a given degree of depletion is less straightforward, especially in the presence of noise and background.

Here we present a general method to achieve, in principle, arbitrary spatial resolution, which relaxes the condition of sequential fluorescence emission. We encode information about the fluorophores nanometer scale spatial distribution within the temporal dynamics of the fluorophore’s transition and decode it using a fast and robust phasor approach. Since the encoding procedure uses temporal modulation of the sample illumination, the method also isolates any uncorrelated background signal. For example, by generating controlled gradients in the fluorophore’s (singlet) excited-state lifetime via SE, we demonstrate that this method provides background-free nanometer scale imaging of the subcellular structures. This approach opens a new route for super-resolution microscopy based on the encoding and decoding of spatio-temporal information through manipulation of the fluorophore dynamics.

## Results

### The SPLIT method

**Figure 1:**
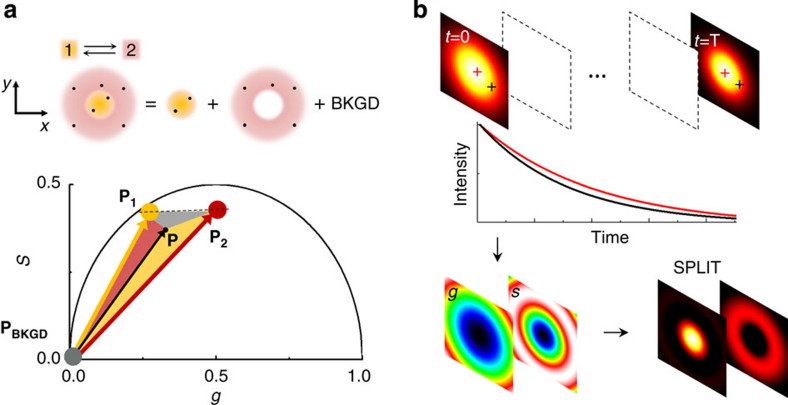
Schematic principle of the SPLIT method. (**a**) It is assumed that the photons are emitted within the DL-PSF with a different dynamics (1 or 2) according to the emitter position. The goal is to separate the photons emitted from 1, those emitted from 2 and those with no temporal dynamics (uncorrelated background, BKGD). This is obtained in the phasor plot expressing the experimental phasor **P** as a linear combination of the phasors **P**_**1**_ and **P**_**2**_ plus the phasor of the background (**P**_**BKGD**_). (**b**) Schematic of the image formation process in SPLIT. The SPLIT method uses the temporal information of the signal at each pixel to generate a set of *g* and *s* images. These images are then processed to obtain the final SPLIT image.

Modulating the sample illumination and measuring the temporal dynamics of the fluorescence can alleviate the stringent condition of silencing all the fluorophores located on a specific portion of the DL-PSF. The *N* photons observed at each pixel can still originate from fluorophores located at any position within the DL-PSF but they could be emitted with different temporal dynamics according to the position of the generating fluorophore in the DL-PSF. The maximum achievable spatial resolution is ultimately determined by the ability to distinguish between different temporal dynamics. The key point here is that the issue of resolving spatial features is translated into the spectroscopy problem of resolving temporal dynamics components. The scheme of the method that we call SPLIT (Separation of Photons by LIfetime Tuning) is depicted in Fig. 1a. Suppose that within the DL-PSF of the microscope, we can distinguish two spatial components 1 and 2 characterized by different temporal dynamics. We make use of the phasor analysis of lifetime data^31^,^32^,^33^ to represent the two different temporal dynamics as two vectors in the phasor plot. The total number of photons detected at one pixel is the sum of the photons originating in the two spatial components plus the uncorrelated background (BKGD) *N*=*N*_1_*+N*_2_*+N*_BKGD_, where only *N*_1_ represents the ‘wanted’ part of all the photons. Following the rules of phasors, the vector **P**=(*g*,*s*) associated with the intensity decay at one pixel can be expressed as the linear combination of the vectors **P**_**1**_=(*g*_1_,*s*_1_) and **P**_**2**_=(*g*_2_,*s*_2_) associated with the two components, with weights *f*_1_ and *f*_2_ given by the corresponding fractions of detected photons: **P**=(*N*_1_**P**_1_+*N*_2_**P**_2_)/*N*=*f*_1_**P**_1_+*f*_2_**P**_2_. This is a linear system of equations in the unknowns *f*_1_ and *f*_2_. We can write this system in the form **P**=*M***f**, where **f**=(*f*_1_,*f*_2_) is the vector of the fractional components and *M*_*ij*_ is the matrix




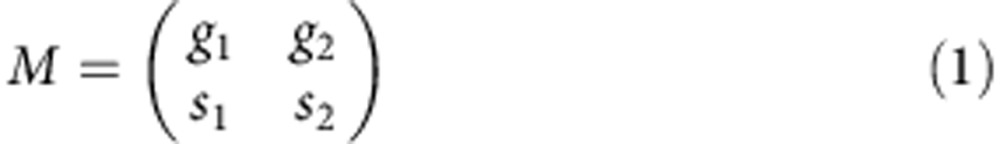




which describes the two different temporal dynamics in the phasor domain. For a given *n* × *n* matrix *M*, the solution of this system is given by **f**=*M*^−1^**P**. Once we find **f** the images *N*_*i*_(*x*,*y*) (*i*=1,…,*n*) of the photons emitted in each of the *n* subdiffraction volumes and the image *N*_BKGD_(*x*,*y*) of the background can be calculated as *N*_*i*_(*x*,*y*)=*f*_*i*_(*x*,*y*)*N*(*x*,*y*) and 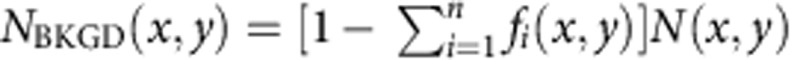. As a result the original image *N*(*x*,*y*) has been split into *n*+1 images based on the assumption that we can observe and distinguish, within our observation volume, *n* different dynamics, associated with *n* linearly independent vectors in the *n*-dimensional phasor space. These dynamics, using the RESOLFT concept^6^, are seen as generalized reversible states of an ensemble of molecules, as they do not correspond necessarily to a specific state of the molecule but rather to a temporal fingerprint. The overall dynamics observed in the DL-PSF is described here using the linear combination properties of phasors: if we assume that there are only two components, the phasor will fall on the line connecting the phasors from pure components (**P**_**1**_ and **P**_**2**_). If we take into account the uncorrelated background as a third component, the phasor **P** will fall in a triangle where the vertices are the phasors **P**_**1**_ and **P**_**2**_ and the phasor **P**_**BKGD**_ of the uncorrelated background (Fig. 1a). To separate more than two dynamics components (*n*>2) and the uncorrelated background, we extend the analysis to phasors obtained at multiple harmonic frequencies^34^,^35^. The image formation process in SPLIT is depicted schematically in Fig. 1b. Shortly, the temporal information of the signal at each pixel is used to generate the *g* and *s* images. These images are then processed to obtain the final SPLIT images.

### The SPLIT method in time-resolved CW-STED

**Figure 2:**
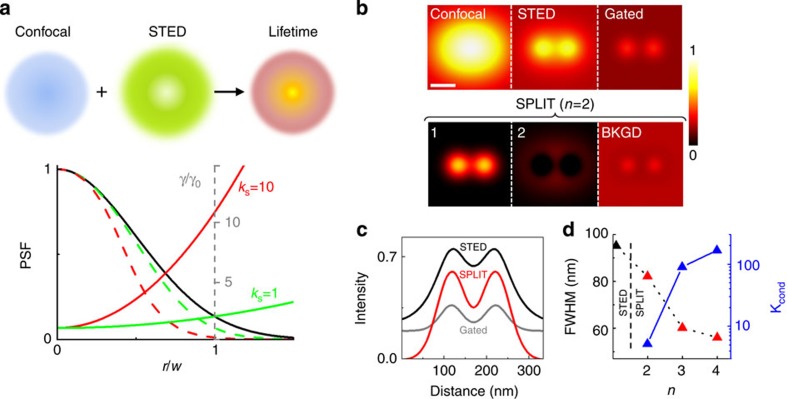
The SPLIT method in time-resolved CW-STED. (**a**) A doughnut-shaped STED beam overlapped with a confocal spot generates a continuous distribution of dynamics within the DL-PSF. The STED beam intensity determines the relative variation of decay rate *γ*/*γ*_0_ (solid green (*k*_S_=1) and red (*k*_S_=10) line) within a Gaussian DL-PSF (solid black line) or the corresponding E-PSF (dashed green (*k*_S_=1) and red (*k*_S_=10) line). (**b**,**c**) Simulated average time-resolved confocal and STED images of two point-like particles plus a uniform level of uncorrelated background (confocal FWHM=200 nm, particles distance=104 nm, *k*_S_=10, *τ*_0_=2.5 ns, *S*=10^5^, *B*=10^4^) and horizontal profile. In the time-gated STED image (*T*_g_=*τ*_0_) the signal becomes very low compared with the background level. In the SPLIT series, the photons of the super-resolved component 1 are efficiently separated from component 2 and from the background. The colourmap represents the simulated intensity normalized to the maximum value of the confocal image. Scale bar, 100 nm. (**d**) Resolution and noise propagation in the SPLIT method versus the number of components. Resolution and noise are quantified, respectively, as the FWHM of the SPLIT E-PSF and the condition number *k*_cond_ obtained for *k*_S_=10 (FWHM of the STED E-PSF is shown for comparison as the first point).

We focus now on the specific case of SE-induced lifetime variations and on the CW-STED microscopy architecture, that is, a Gaussian excitation beam and a doughnut-shaped STED beam (Fig. 2a). However, the proposed approaches can be easily adapted to other configurations and other state transitions. The first ingredient is a model to describe the *n* dynamics components into which to split the measured intensity pixel-by-pixel, namely, we need the matrix *M*. For simplicity, we assume (i) a Gaussian profile of the conventional DL-PSF *h*(*x*′*,y*′*,z*′)=exp(−2*r*^2^/*w*^2^)exp(−2*z*′^2^/*w*_z_^2^), with *w* and *w*_z_ being the beam waists along the radial and axial direction, respectively; and *r*^2^=*x*′^2^+*y*′^2^ the radial distance from the focal point (*x*=0, *y*=0) (ii) a parabolic approximation for the doughnut-shaped STED beam *I*_STED_(*r*)=*I*_STED_(*w*)*r*^2^/*w*^2^, with *I*_STED_(*w*) the STED beam intensity at position *r*=*w*; (iii) a single exponential decay rate for the unperturbed fluorophores *γ*_0_=1/*τ*_0_, where *τ*_0_ is the unperturbed excited-state lifetime. Under these assumptions the spatial distribution of the decay rate is approximated by a parabolic function *γ*(*r*^2^)=*γ*_0_+*γ*_0_*k*_S_*r*^2^/*w*^2^, where *k*_S_=*I*_STED_(*w*)/*I*_SAT_ is the ratio between *I*_STED_(*w*) and the saturation value *I*_SAT_ for which the probability of decay due to SE and spontaneous emission are equal (see Supplementary Note 1). Importantly, the value of *k*_S_ determines the relative variation of decay rate values within the E-PSF of the CW-STED microscope (Fig. 2a).

The time-dependent fluorescence signal *F*(*x*,*y*,*t*) at each pixel can be obtained by integrating the contribution of all the fluorophores located in the E-PSF centred in the pixel position (*x*,*y*) (see Supplementary Note 1)




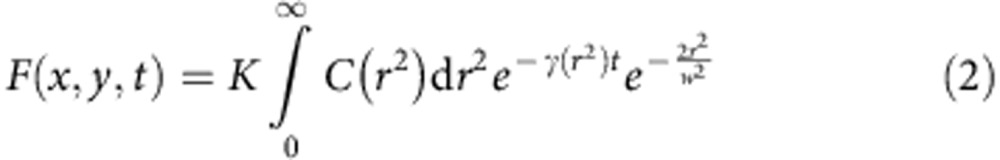




where *C*(*r*^*2*^) describes the concentration of the fluorophores in a concentric region of radius *r* around the pixel position and *K* is a constant that depends on the quantum yield of the fluorophore, the maximum of the excitation intensity and the detection efficiency. In contrast to deconvolution methods, the proposed method does not try to reassign photons to the original position, thereby the position at which the fluorophores are located within each concentric cylinder is not important. In other words, this approach does not need prior knowledge of the E-PSF of the CW-STED system, which makes this approach suitable also for non-expert users. The temporal dynamics of *F*(*x*,*y*,*t*) encodes nanoscale spatial information in the distribution of exponential decay components. We split the integral and calculate *n* dynamics components defined uniquely by the parameters *γ*_0_ and *k*_S_ (see Methods), from which the decoding matrix *M* is derived.

We tested the proposed method on synthetic time-resolved CW-STED images obtained with known *γ*_0_ and *k*_S_. Figure 2b shows the ability of the SPLIT method in separating the photons coming from the inner subdiffraction volume from those of the periphery and the uncorrelated background, whereas time gating is affected by an increasing fraction of background (Fig. 2b,c). Notably, the spatial features appearing on the background image are due to the approximation of the continuous distribution of dynamics to only two components (see Supplementary Fig. 1). The spatial resolution of the SPLIT image can be further increased using a higher number *n* of components (Fig. 2d and Supplementary Fig. 2a). In gated CW-STED microscopy this is done by increasing the time-delay *T*_g_ (Supplementary Fig. 2b). For instance, using *n*=4 it is possible to get the same spatial resolution of CW-STED but at a STED beam intensity, which is 1 order of magnitude lower (Supplementary Fig. 2c). Note that the separation of dynamics obtained in the SPLIT method is conceptually different from time gating. The separation in SPLIT is based on the analysis of variations of the signal over the whole time range (Fig. 1b). For this reason, a SPLIT image could be obtained at increasing values of *n* even when *T*_g_ is limited by the period *T* (the reciprocal of the repetition rate, typically in the order of 10^7^ Hz) (Supplementary Fig. 2d).

The SPLIT image exploits the additional spatial information potentially encoded in the *g*(*x*,*y*) and *s*(*x*,*y*) images (see Supplementary Note 2). This additional amount of spatial information is available on a STED image but not on a confocal image (Supplementary Fig. 3). The improvement in spatial resolution in a SPLIT image at increasing values of *n* comes from the analysis of increasingly higher temporal frequencies in the signal (see Supplementary Note 2). Thus, it comes from a better sorting of photons as a function of dynamics/locations. However, when noise is taken into account, the larger the number *n* of components the higher will be the noise propagated to the final images, quantified as the condition number *k*_cond_ of the matrix *M* to invert (Fig. 2d and Supplementary Fig. 4; see Supplementary Note 3). For a given level of depletion and for a given level of noise, there is a finite number of values of *n* for which the noise in the final image is below a desired threshold.

### Experimental determination of unknown decoding parameters

**Figure 3:**
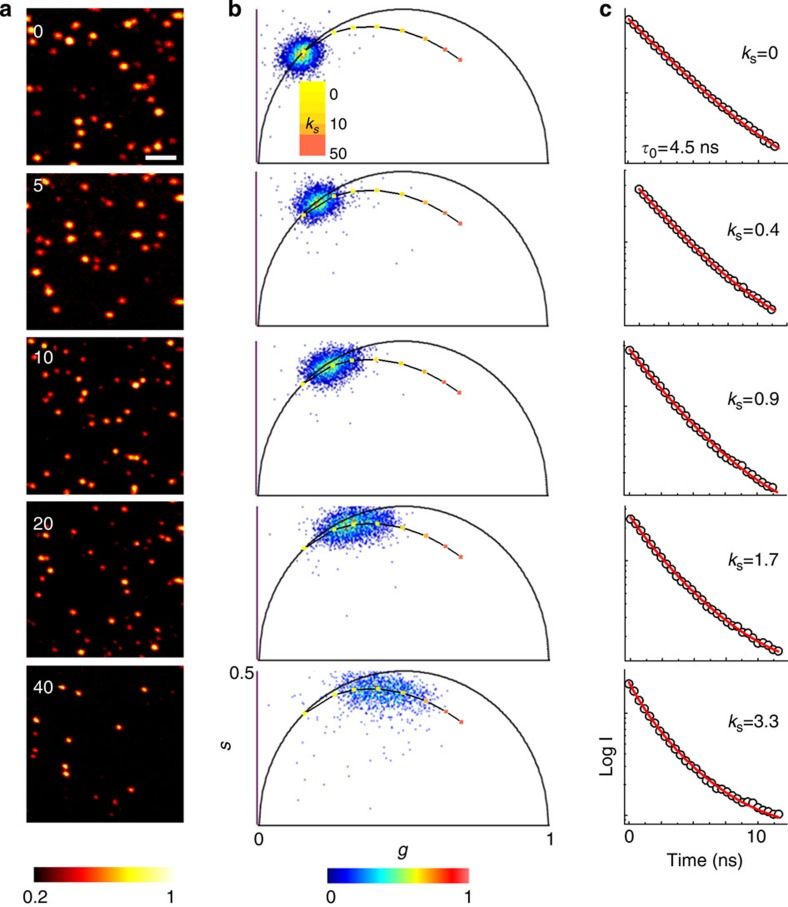
STED phasors and average dynamics at different STED powers. (**a**) Time-resolved STED images of 40 nm yellow–green fluorescent beads at several STED beam powers. Numbers indicate STED beam power in mW (measured at the back aperture of the objective lens). The colourmap in **a** represents the time-integrated intensity detected at one pixel normalized to the maximum value of each image (threshold set to 20% of the maximum value). (**b**,**c**) Phasor plots (**b**) and average time-resolved decays (**c**) associated to the images in **a**. Increasing STED powers induce an increasing spread of the phasor and an increasing stretching of the average decay from an exponential (*k*_S_=0, *τ*_0_=4.5 ns) into the trend described by equation (3) with *k*_S_>0. This equation describes a trajectory in the phasor plot for increasing values of *k*_S_ (solid line), which overlaps with experimental phasor. The values of *k*_S_ obtained from the fit of the average decay scale linearly with the STED beam power. Scale bar, 1 μm.

To decode the spatial information hidden in the gradients of dynamics induced by the STED beam, we need to know, according to our model, only the two parameters *γ*_0_ and *k*_S._ The parameter *τ*_0_=1/*γ*_0_ is usually known for a specific fluorophore or can be easily measured from the sample with the very same instrumentation by setting the STED beam power to zero. The parameter *k*_S_=*I*_STED_(*w*)/*I*_SAT_ is proportional to the STED beam power but its precise value depends on the optical configuration and on the properties of the sample. It is interesting that, using our analytical model of the SE-induced lifetime variations, we are able to estimate the value of *k*_S_ from the same image *F*(*x*,*y*,*t*) by considering the average time-resolved decay of all the pixels of an image (see Supplementary Note 1),




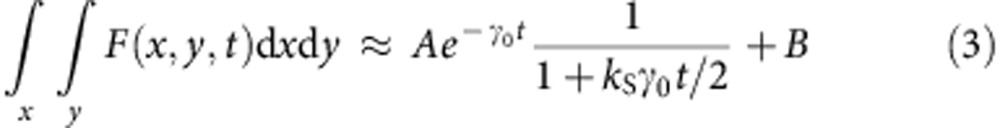




where *B* denotes the uncorrelated background. To validate the model, we imaged 40 nm fluorescent beads at several STED beam powers (Fig. 3a). The two-dimensional (2D) histogram of the values *g*(*x*,*y*) and *s*(*x*,*y*) associated with each pixel is represented in the phasor plot (Fig. 3b). The phasor of the confocal image (zero STED power) is centred to the position corresponding to a single exponential decay with *τ*_0_=4.5 ns. The same value *τ*_0_=1/*γ*_0_ is found by fitting the average photon-arrival time histogram to equation (3) with *k*_S_=0 (Fig. 3c). With the increasing of the STED beam power the phasor becomes elongated as different dynamics are sampled in the image. The precise value of *k*_S_ at each STED power can be determined by fitting the average photon-arrival time histogram to equation (3) with *τ*_0_ fixed (*τ*_0_=4.5 ns). The good agreement with the model is confirmed by the linearity between *k*_S_ and the STED beam power. The phasor associated with the theoretical decay expressed by equation (3) for *τ*_0_=4.5 ns and increasing the value of *k*_S_ describes the expected trajectory of the average phasor of the image as a function of the STED power. To assess the validity of the method for the imaging of non-point-like structures, we also performed simulations using more convoluted structures similar to those found in cytoskeletal networks (see Supplementary Fig. 5). Also in this case, by using the values of *k*_S_ obtained by fitting the average time-resolved STED decay of the image, we were able to separate the images of the super-resolved components and the background.

### SPLIT imaging of subcellular structures

**Figure 4:**
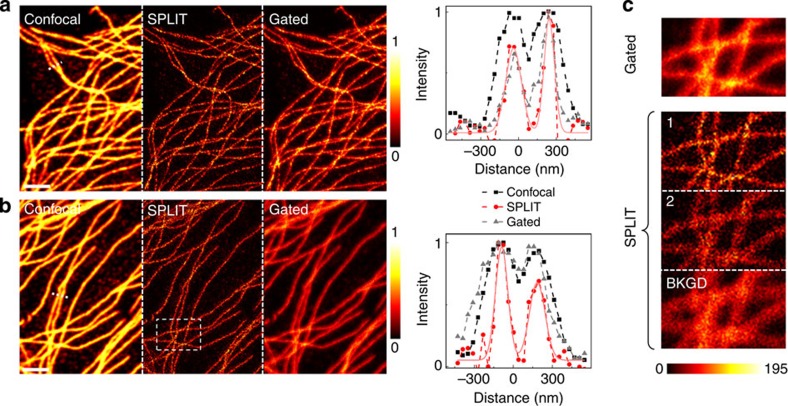
Application of the SPLIT method to biological imaging. (**a**,**b**) Microtubules in fixed HeLa cells labelled by immunocytochemistry with the organic dyes Alexa Fluor 488 (**a**) and Oregon Green 488 (**b**). Shown are the confocal image, the SPLIT (*n*=2, first component) image, the time-gated image (*T*_g_=1 ns) and the intensity profile along the dashed line. The colourmap represents the fluorescence intensity normalized to the maximum value of each image. (**c**) The time-gated image is compared with the full SPLIT series (*n*=2) for the region highlighted in **b**. The colourmap represents the fluorescence intensity expressed in counts per 0.1 ms. The STED beam power (measured at the back aperture of the objective lens) was *P*_STED_=40 mW. Scale bars, 2 μm.

We finally applied the SPLIT method to the imaging of biological structures, namely microtubules on fixed HeLa cells, as reported in Fig. 4. We compare results obtained by labelling tubulin with two different dyes, Alexa Fluor 488 (Fig. 4a) and Oregon Green 488 (Fig. 4b). The parameters *γ*_0_ and *k*_S_ are found from the same experimental time-resolved data sets by fitting the average decay of all the pixels (Supplementary Fig. 6). According to the model and the simulations, the expected full-width at half-maximum (FWHM) of the SPLIT image (*n*=2) is of the order of 100 nm, as confirmed by the experimental intensity profiles (Fig. 4 and Supplementary Fig. 7). The SPLIT image is compared with the confocal and the gated CW-STED image (gating time is set to *T*_g_=1 ns), showing the improvement in spatial resolution with simultaneous efficient removal of background. Under the same experimental conditions (*λ*_STED_=560 nm) the Oregon Green 488 fluorophores exhibit more uncorrelated background due to STED beam-induced excitation, thereby in the relative gated STED image, the improvement in resolution is totally masked by the strong background induced by direct STED beam excitation. Even though specific methods for background subtraction^27^,^28^,^29^ have been developed recently, it is remarkable how the uncorrelated background photons are automatically separated in the calculation of the SPLIT image (Fig. 4c). This is possible because we are operating a separation of the signal in the frequency domain (where the uncorrelated background is well-separated from the other components) rather than in the time domain (where the uncorrelated photons are evenly distributed). Here in addition to the different temporal dynamics of the excitation and STED beams, sufficient to remove the uncorrelated background, we are also decoding the spatial frequencies hidden in the gradient of dynamics induced by the STED beam.

## Discussion

We have shown that super-resolution can be achieved by proper spatial tuning of the fluorophore signal dynamics as a function of their position within the detection volume of a scanning microscope. A key element of this method is the phasor representation of the signal dynamics, which provides a procedure to recover the hidden spatial information lost during the time-averaging process. To the best of our knowledge, this is the first time that a tool like the phasor, originally developed for spectroscopy, has been applied for imaging to increase its spatial resolution beyond the diffraction limit.

In particular, we have described a robust, fit-free and user-friendly method for separating the different dynamics components of a time-resolved CW-STED measurement and isolating the high spatial resolution components, even in the presence of uncorrelated background. The method is ultimately limited by the noise in the measurement of the dynamics but, importantly, we can predict the maximum number of components that we can resolve into for a given level of noise. The spatially associated dynamics components are generated starting from only two parameters (*γ*_0_ and *k*_S_) that can be easily assessed in the same image. Notably, these parameters alone are sufficient to decode the subdiffraction spatial information without prior calibration of the confocal PSF.

This approach is different from other super-resolution approaches making explicit use of the temporal dynamics of fluorescence. For instance, in the DSOM implementation, which exploits a singlet–triplet-state transition, the contribution of the faster decaying fluorophores in the centre of the DL-PSF is extracted by fitting the signal at each pixel with a multi-exponential model. In time-gated STED, the size of the E-PSF is reduced by restricting detection to late photons, an approach that is of straightforward operation but not equivalent to resolving dynamics components. Indeed, in time-gated STED the subtraction of background requires further analysis, and, worth noting, the information hidden in the early photons is lost. In the Gaussian STED beam implementation, instead of performing spectroscopy on each pixel of the image, an image is associated to each bin of the time decay and a super-resolved image is obtained from a reconstruction using a suitable linear combination of these images. Importantly, the derivation of the optimal coefficients for the linear combination needs the knowledge of the E-PSF of the Gaussian STED implementation.

When the E-PSF of the imaging system is known, a powerful approach to improve the spatial resolution is image deconvolution^36^. Generally speaking, deconvolution uses the fact that each pixel of an image encodes spatial information of structures contained in the neighbouring pixels. Given the E-PSF of the system, a deconvolution algorithm tries to recover such information. In comparison, our SPLIT approach does not use the *a-priori* spatial information provided by the E-PSF, which is unfortunately not straightforward information. However, it is clear that the spatial information provided by the E-PSF and the temporal information provided by the signal dynamics can work in synergy. In particular, one could develop a dedicated deconvolution algorithm which uses a spatio-temporal E-PSF^26^, or apply a conventional spatial deconvolution algorithm on the SPLIT image. Indeed, the SPLIT method is a linear and space-invariant system, thus fully characterized by an E-PSF (Supplementary Note 2).

Compared with all these methods, the SPLIT approach appears as a simple and efficient way to extract the high spatial frequencies encoded in the variations of fluorescence and the only method to operate the decoding explicitly in the frequency domain in a pixel-by-pixel manner. SPLIT exploits the additional spatial information potentially encoded in the *g*(*x*,*y*) and *s*(*x*,*y*) images describing the evolution of the fluorescence signal at each pixel.

It is worth noticing that the generality of the method is not compromised by the specific assumptions used to simplify the analytical description. For instance, even though we have considered lifetime variations generated along only the radial direction but not along the axial, the model can be easily adapted to take into account decay rate gradients generated by 3D-structured STED beams^37^. We have also assumed that unperturbed temporal dynamics could be described by a single exponential decay, though for many fluorophores this is not true. However, one of the major advantages of phasor analysis is precisely that both exponential and non-exponential decays are equally described as vectors in the phasor space, and it is on these vectors that we perform our analysis.

In STED microscopy, lifetime tuning and photon separation can already be achieved at low-intensity illumination and fast acquisition speed, which are among the key requirements for non-invasive live cell imaging. The fact that photons are spatially sorted and not suppressed makes it quite intriguing to explore this method in single-molecule techniques like fluorescence correlation spectroscopy^38^, where a high SNR is generally required and where STED beam-induced background appears as an obstacle towards full 3D nanoscale fluctuation imaging^39^. The method is not limited to SE but can be applied to other types of dynamics or spectra in general, for instance the transition dynamics of reversibly photoswitchable proteins^40^,^41^ or, similar to DSOM, the singlet-to-triplet states dynamics. As long as the observed dynamics occur on a faster time scale compared with the pixel dwell time, the method has no limitations in terms of speed. Its resolution performances will be ultimately determined by our ability in generating and discriminating different dynamics components against the noise. This suggests looking for those physical phenomena that maximize transient dynamics or spectral gradients within the DL-PSF rather than decrease the total number of emitted photons. In this perspective, an interesting point for future developments will be the design of optical probes whose photophysical or spectral properties could be easily modulated without detrimental effects. We envisage that additional advantages could be gained by exploiting the more complex dynamics of photophysical systems involving a larger number of molecular states^42^. With a rapidly evolving imaging technology helping us to improve sensitivity and thereby SNR, we expect the next generation of fast, background-free optical super-resolution microscopy to make extensive use of spatio-temporal information encoding strategies.

## Methods

### Calculation of phasors and their linear combination

The phasor coordinates at a harmonic *h* corresponding to the time-resolved intensity *I*(*t*) are defined as^34^:




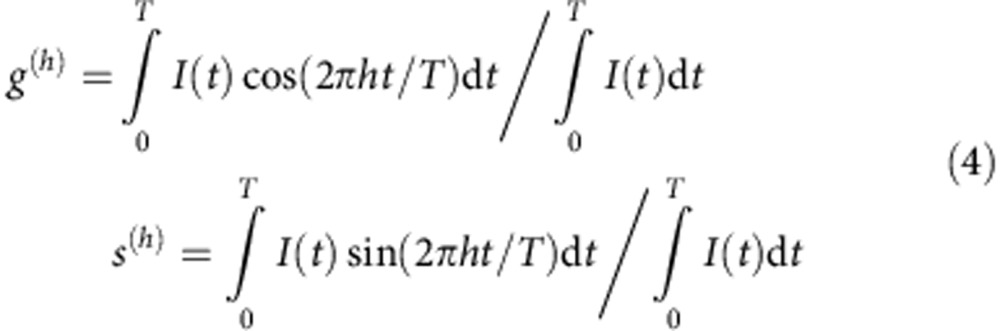




where *T* is the period of excitation or a smaller value for which the function *I*(*t*) has already decayed to the uncorrelated background value. If the intensity at one point is due to the sum of two components plus the uncorrelated background, *I*(*t*)=*I*_1_(*t*)+*I*_2_(*t*)+*I*_BKGD_, then its phasor can be expressed as a linear combination of the phasors of the two components and the phasor of the background (that is, a null vector):




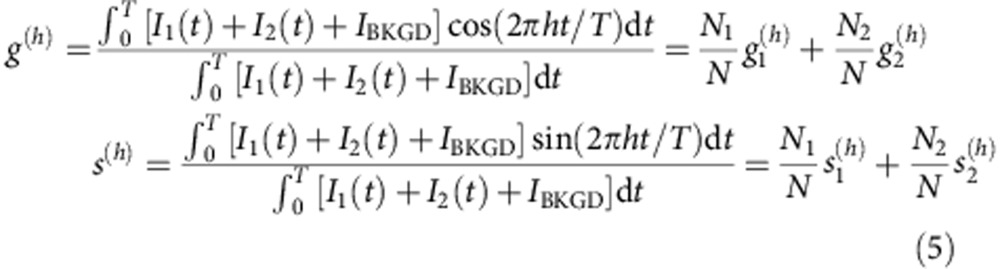




where the total number of photons *N* detected at one pixel is the sum of the photons originating in the two spatial components plus the uncorrelated background *N*=*N*_1_*+N*_2_*+N*_BKGD_. It can be seen that since the uncorrelated background is independent of *t*, its phasor coordinates are (0,0). The addition of uncorrelated background does not affect the value of the phase *φ*=tan^−1^(*s*/*g*) but decreases the value of modulation *m*=(*g*^2^+*s*^2^)^1/2^ of a phasor.

### Separation of the intensity into *n* components

The calculation of an arbitrary number of fractional components *n* was obtained by considering a matrix-vector representation and extending the phasor analysis to higher harmonics. If the intensity *I*(*t*) is sampled in *N*_bin_ time windows, then the maximum number of harmonics we can use is *N*_bin_/2. **P**=(*g*, *s*, *g*^(2)^, *s*^(2)^,…) is the *n*-element vector formed by the phasor coordinates derived from the intensity decay at one pixel. The last element of the vector **P** is *g*^((*n*+1)/2)^ (if *n* is odd) or *s*^(*n*/2)^ (if *n* is even). Provided that we know the temporal dynamics of the *n* components *I*_*j*_(*t*), we defined *M*_*ij*_ as the *n* × *n* matrix whose column *j* is the vector with the phasor coordinates of the *j*^*th*^ component up to *g*_*j*_^((*n*+1)/2)^ (if *n* is odd) or *s*_*j*_^(*n*/2)^ (if *n* is even):




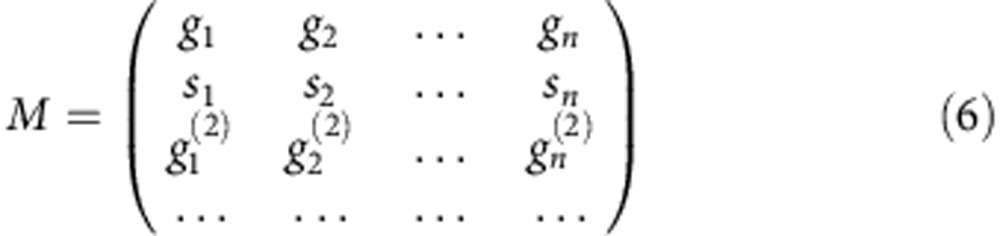




Then, provided that det *M*≠0, the *n*-elements vector of the fractional components **f**=(*f*_1_,…,*f*_*n*_) was calculated by **f**=*M*^**−1**^**P**.

### Calculation of dynamics components in time-resolved CW-STED

In time-resolved CW-STED microscopy, the exact temporal dynamics of *F*(*x*,*y*,*t*) depends on the function *C*(*r*^2^), which acts as a pixel-dependent weight on the exponential decay components exp(−*γ*(*r*^2^)*t*). To approximate the continuous distribution of decays in a discrete number *n* of components, we split the integral into *n* parts




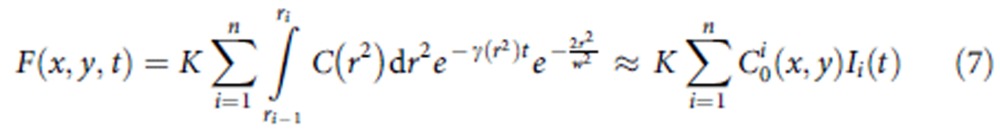




where *I*_*i*_(*t*) describes the average dynamics of the *i*th component




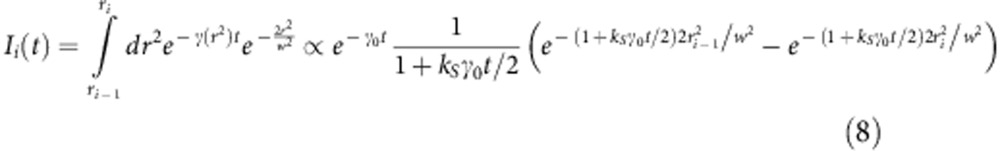




The boundaries *r*_*i*_ of the subdiffraction volumes were chosen in such a way that, for *C*(*r*^2^)=constant, all the time-correlated photons were split in equal number among the *n* components (see Supplementary Note 1).

### Simulations and data analysis

Simulations of time-resolved STED microscopy images of point-like particles were performed using custom-built software in ImageJ. The value of the intensity at pixel (*x*,*y*) and time *t* originating from *N*_p_ point-like particles was set as: 
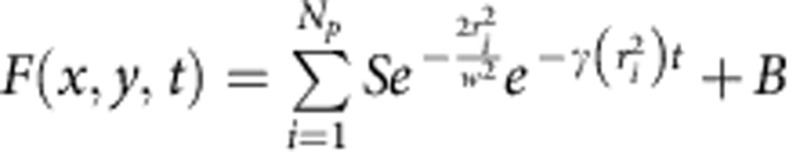
 where 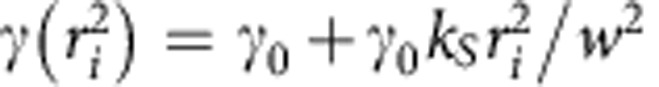, *γ*_0_=1/*τ*_0_ is the spontaneous decay rate and *r*_*i*_^2^ is the square of the distance from the *i*th particle. Stacks consisted of 128 frames of 64 × 64 pixels. The parameter *S* is the maximum intensity signal from a particle expressed in counts detected at one pixel in one frame of the stack. The parameter *B* represents a uniform level of background expressed in counts detected at one pixel in one frame of the stack. The resulting ideal image is successively corrupted by Poisson noise. For all the simulations reported in Fig. 2b, Supplementary Fig. 1, Supplementary Fig. 2, Supplementary Fig. 3 and Supplementary Fig. 4 the confocal waist was set to the value *w*=167 nm (corresponding to a FWHM=200 nm), the pixel size to 5.2 nm and the time step to Δ*t*=0.097 ns. The other parameters were varied as indicated in the figures. The confocal image was obtained by adding all the frames of the confocal stack (*k*_S_=0). The STED image was obtained by adding all the frames of the STED stack (*k*_S_>0). The time-gated STED image was obtained by adding only those frames of the STED stack for which *t*≥*T*_g_.

Simulations of convoluted structures similar to those found in cytoskeletal networks (Supplementary Fig. 5) were performed using MATLAB (MathWorks). The cytoskeletal phantom was composed of 75 filaments with diameter of 30 nm. To each filament, we associated a value between 0 and 1 to simulate differences in the brightness of the structures. The maximum total number of photons detected from a single pixel position in one frame of the stack was set to *S*=120. A uniform level of background was set to the value *B*=0.5 counts per each pixel and per each frame of the stack. The phantom was convolved with a theoretical 3D (*x*,*y*,*t*) E-PSF of a CW-STED microscope^26^ and the obtained image was corrupted by Poisson noise. In the example reported in Supplementary Fig. 5, the stack consisted of 64 frames of 256 × 256 pixels, showing an area of 10 × 10 μm. The confocal FWHM was set to 235 nm. The total time period was set to *T*=12.5 ns so that Δ*t*=12.5/64 ns. The unperturbed decay rate was set to *τ*_0_=3.15 ns and relative variation of decay rate was set to the value of *k*_S_=12.7. Again, in this case, the STED image was obtained as the sum of all the frames in the stack. For the simulations of cytoskeletal structures the value of *γ*_0_ was known, whereas the value of *k*_S_ was determined by fitting equation (3) to the average time-resolved STED decay of the image (Supplementary Fig. 5).

The parameters *γ*_0_ and *k*_S_ relative to the experimental biological images were extracted from the full fields of view reported in Supplementary Fig. 6. We extracted first the value of *γ*_0_ by fitting equation (3) to the average confocal decay by fixing *k*_S_=0. Then we extracted the value of *k*_S_ by fitting equation (3) to the average STED decay. By fitting the confocal decay to a single exponential decay, we obtain the value of *τ*_0_ (*τ*_0_=2.7 ns for the Alexa Fluor 488 sample; *τ*_0_=1.8 ns for the Oregon Green sample). Then we fix this parameter and estimate *k*_S_ from the fitting of the STED decay (*k*_S_=7.8 for the Alexa Fluor 488 sample; *k*_S_=4.9 for the Oregon Green sample). To test if the parameter *k*_S_ varied across the sample, we performed the same analysis in smaller regions-of-interest of different size (Supplementary Fig. 6c). The values of *k*_S_ extracted from regions-of-interest of size down to about 32 pixels were quite consistent between different regions and consistent with the *k*_S_ value extracted from the whole image. We used the parameters *γ*_0_ and *k*_S_ to split the time-resolved STED image into the super-resolved components (1 and 2) and the background (BKGD).

The SPLIT analysis was implemented writing a custom code in MATLAB. For each pixel (*x*,*y*) of the time-resolved STED image, the phasor coordinates *g*(*x*,*y*) and *s*(*x*,*y*) were calculated using a FFT algorithm. The phasor plots reported in Fig. 3 are the 2D histograms of these values and were obtained using Globals for Images (Laboratory for Fluorescence Dynamics). The values of *γ*_0_ and *k*_S_ were used to generate the expected theoretical decays of the *n* spatial components and the decoding matrix *M*. The condition number *k*_cond_ of the matrix *M* was calculated in MATLAB.

All the fitting procedures were performed in OriginPro (OriginLab) using an unweighted least squares procedure.

### Experiments

All the time-resolved CW-STED experiments were performed on a home-built CW-STED microscope^25^,^29^. The excitation beam was provided by a supercontinuum source and the STED beam was provided by a CW visible fibre laser (VFL) emitting at 560 nm (VFL-P-1000-560, MPB Communication Inc.). We generated the supercontinuum source by pumping a photon-crystal-fibre (femtoWHITE-800, NKT Photonics) with a femtosecond mode-locked Ti:Sapphire laser of 150 fs pulse width, 80 MHz repetition rate (Chameleon, Vision II, Coherent). To obtain a doughnut-shaped diffraction pattern at the focus the STED beam passed through a polymeric mask imprinting 0–2*π* helical phase-ramps (VPP-A1, RPC Photonics). The STED and the excitation beams were collinearly aligned using two dichroic mirrors (zt-488-RDC and z-560-sprdc, AHF analysentechnik), then deflected by two galvanometric scanning mirrors (6215HM40B, CTI-Cambridge) and directed towards the objective lens (HCX PL APO 100/1.40/0.70 Oil, Leica Microsystems) by the same set of scan and tube lenses as the ones used in a commercial scanning microscope (Leica TCS SP5, Leica Microsystems). The fluorescence light was collected by the same objective lens, de-scanned and passed through the dichroic mirrors as well as through a fluorescence band pass filter (ET Bandpass 525/50 nm, AHF analysentechnik) before being focused (focal length 60 mm, AC254-060-A-ML, Thorlabs) into a fibre pigtailed single photon avalanche diode (PDF Series, Micro Photon Devices). Photon-arrival times were detected at each pixel by a time-correlated-single-photon-counting-card (SPC-830, Becker & Hickl). Synchronization was obtained from the reference signal provided by the Ti:sapphire laser. All imaging operations were automated and managed by the software Imspector (Max Planck Innovation). For both the STED and excitation light, the average power *P* was measured at the back aperture of the objective lens. Due to losses in the objective lens, the power at the sample is actually lower by 15% and 12% at 488 nm and 560 nm, respectively.

Samples of 40-nm diameter yellow–green fluorescent spheres (Yellow–Green, Invitrogen) were prepared as follows. The spheres were diluted in water by 1:3,000 (v/v). We dropped the dilute solution of fluorescent beads onto a poly-L-lysine (Sigma) coated glass coverslip, waited 10 min, washed it with water and dried the coverslip by blowing nitrogen onto it. Finally we mounted the coverslip with a special medium (Mounting Medium, Invitrogen) and we observed with the STED microscope.

For immunofluorescence imaging, HeLa cells were cultured on glass coverslips (18-mm diameter) in Dulbecco’s modified Eagles medium (Invitrogen) supplemented with 10% foetal bovine serum (Invitrogen), 100 IU ml^−1^ penicillin and 100 μg ml^−1^ streptomycin (Invitrogen) at 37 °C in a humidified atmosphere containing 5% CO_2_ for 24 h. Plated cells were rinsed with phosphate-buffered saline (PBS) (0.1 M, pH 7.4) and fixed by incubation in 4% formaldehyde in PBS for 15 min. Fixed cells were washed with PBS and permeabilized for 30 min at room temperature with 3% normal bovine serum albumin and 0.1% Triton X-100 in PBS. The cells were then incubated with the monoclonal mouse anti-α-tubulin antiserum (Sigma Aldrich) diluted in 3% bovine serum albumin 0.1% Triton/PBS (1:1,000) for 1 h at room temperature. Anti-α-tubulin antibody was revealed using Alexa Fluor 488 goat anti-mouse IgG (1:500, Molecular Probes) or Oregon Green 488 goat anti-mouse IgG (1:500, Molecular Probes). The coverslips were rinsed in PBS and then placed in an open-bath imaging chamber containing PBS and observed with the STED microscope.

## Additional information

**How to cite this article:** Lanzanò, L. *et al.* Encoding and decoding spatio-temporal information for super-resolution microscopy. *Nat. Commun.* 6:6701 doi: 10.1038/ncomms7701 (2015).

## Supplementary Material

Supplementary Information Supplementary Figures 1-7, Supplementary Notes 1-3 and Supplementary References
