# Supplementary material for: Encoding and decoding spatio-temporal information for super-resolution microscopy
Source: Nat Commun. 2015 Apr 2;6:6701. doi: 10.1038/ncomms7701 (PMC4384168; doi:10.1038/ncomms7701)
Supplement: Supplementary Information — Supplementary Figures 1-7, Supplementary Notes 1-3 and Supplementary References [file ncomms7701-s1.pdf]

Supplementary Information

Supplementary Figures

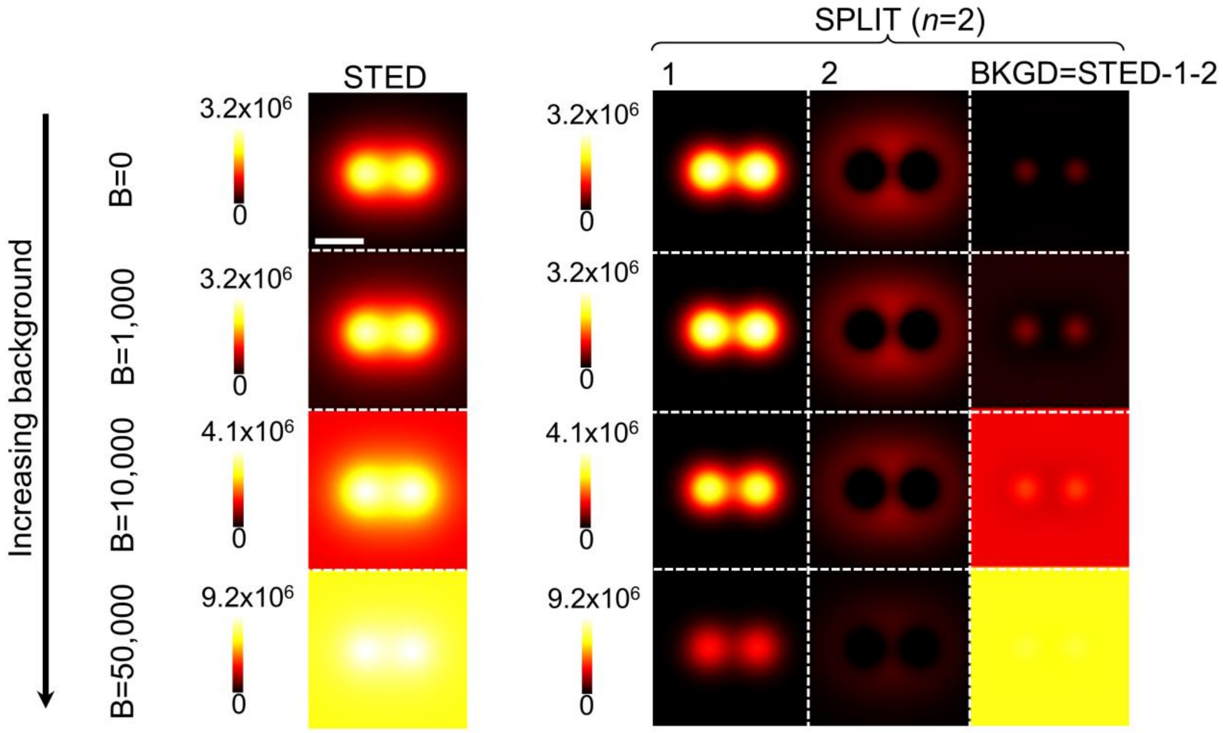

Supplementary Figure 1. SPLIT of simulated data in presence of background. SPLIT method (n = 2) applied to simulated time resolved STED images in presence of an increasing level of background. The parameters were set as follows: confocal FWHM = 200 nm, particle distance = 104 nm, τ_0 = 2.5 ns, k_S = 10, $S = 10^5$. The level of background, uniform for each image, was varied by changing the value of B as indicated in the figure. The spatial features that are visible on the calculated background image, even when the background level is set to B=0, are due to the fact that the sum of 1 and 2 does not correspond to the STED image. This is because we are using only 2 components to approximate the continuous distribution of decays generated by the STED beam. The colormap represents the simulated intensity. Scale bar 100 nm.

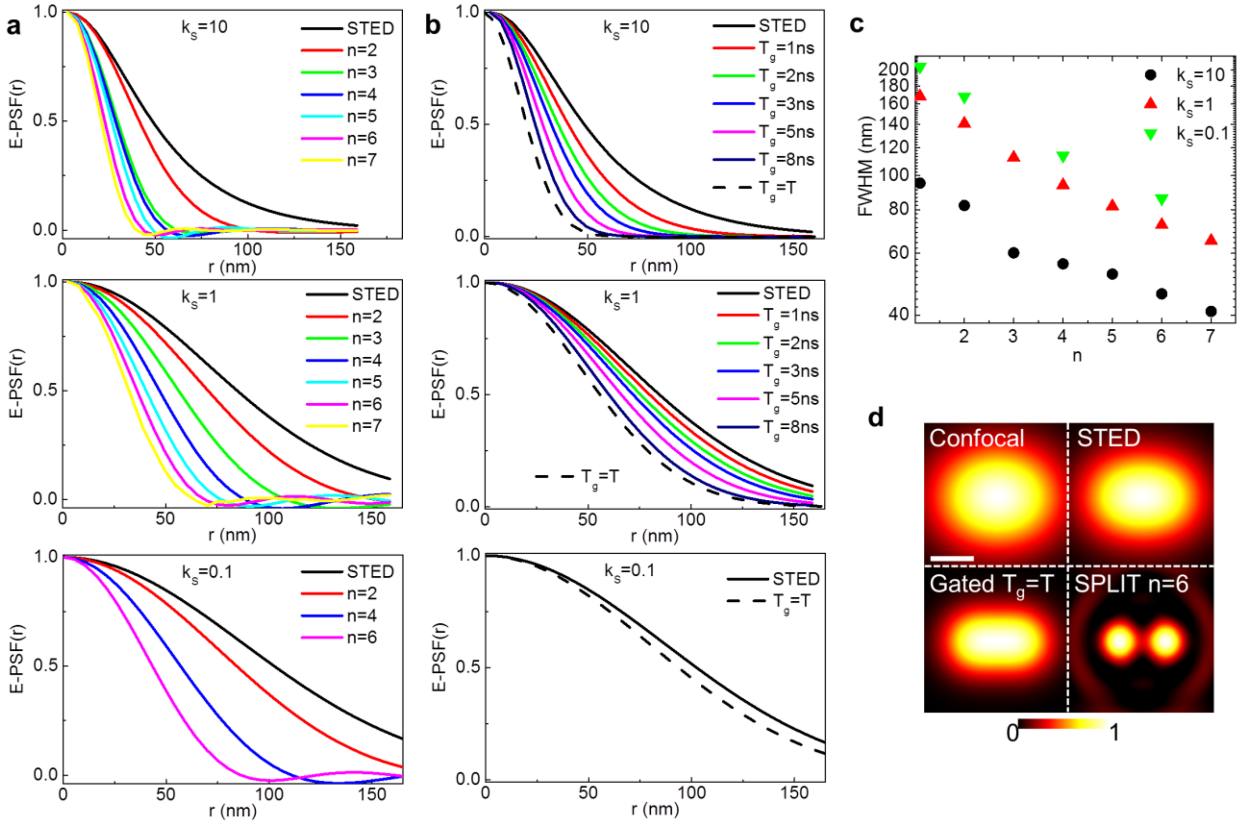

Supplementary Figure 2. Spatial resolution in SPLIT versus time-gated CW-STED. (**a**,**b**) Effective PSF (E-PSF) for the super-resolved SPLIT (**a**) and time-gated (**b**) images obtained from simulations of single point-like particles using the following parameters: a confocal PSF with FWHM = 200 nm, an unperturbed lifetime $\tau_0 = 2.5$ ns, a period $T = 12.5$ ns, $B = 0$, $S = 10^5$ and STED intensity levels $k_S = 10$, $k_S = 1$ and $k_S = 0.1$ respectively. (**c**) The SPLIT FWHM (i.e. the FWHM of the first SPLIT component) is reported as a function of n and compared with the corresponding STED FWHM value (first point). (**d**) Simulation of two point-like particles with the following parameters: confocal FWHM=200 nm, particles distance = 104 nm, $\tau_0 = 4$ ns, $k_S = 1$, $S = 10^{12}$, $B = 0$, $T = 12.5$ns. In this particular example the gated image with maximum resolution is obtained by setting $T_g = T = 12.5$ ns. On the contrary, the resolution of the SPLIT image can be increased by using a higher number n of components (shown is $n = 6$). This example is provided to illustrate that time-gating and SPLIT operate in a different way and doesn't correspond necessarily to a practical case. The colormap represents the simulated intensity normalized to the maximum value of each image. Scale bar 100 nm.

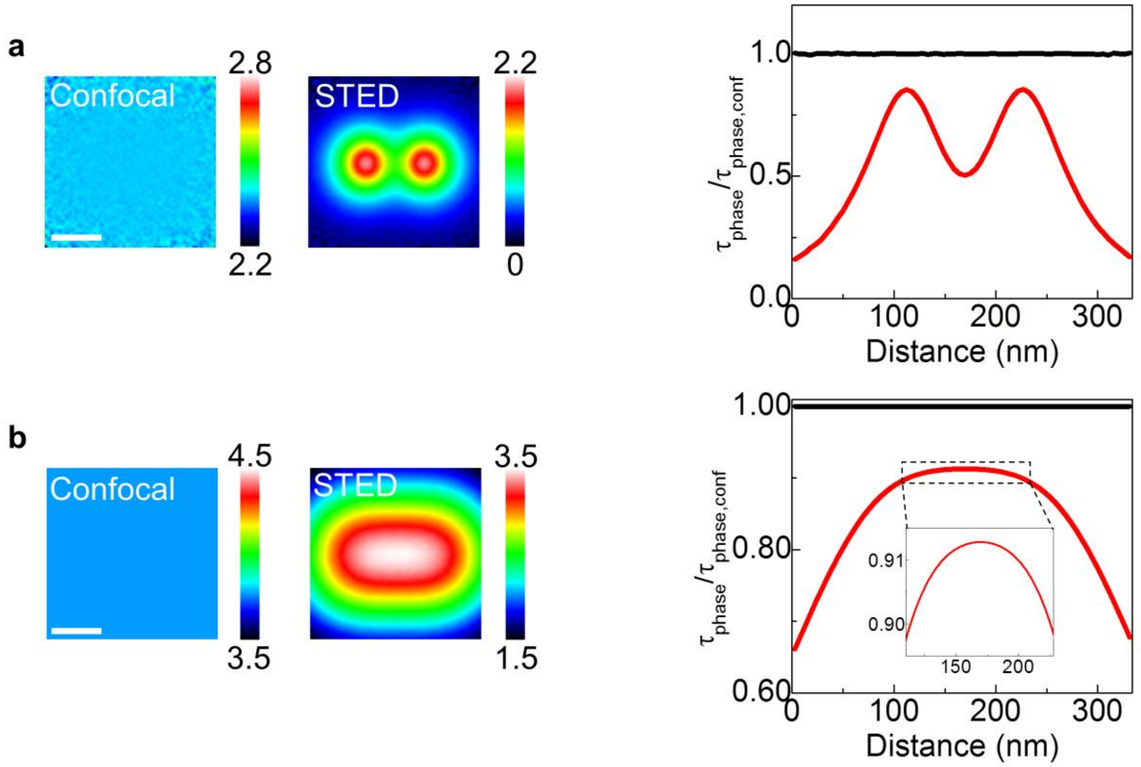

Supplementary Figure 3. Image of the parameter τ_{phase} in confocal and CW-STED. (a,b) Images of the parameter $\tau_{phase}=(T/2\pi)(s/g)$ for confocal versus CW-STED simulated data and horizontal profile. Parameters of the simulation in (**a**): confocal FWHM = 200 nm, particles distance = 104 nm, k_S = 10, τ_0 = 2.5 ns, S = 10^5, B = 10^4 , T = 12.5ns. Parameters of the simulation in (**b**): confocal FWHM=200 nm, particles distance = 104 nm, k_S = 1, τ_0 = 4 ns, S = 10^{12}, B = 0, T = 12.5ns. Colormap represents the value of τ_{phase} in ns. Scale bars 100 nm.

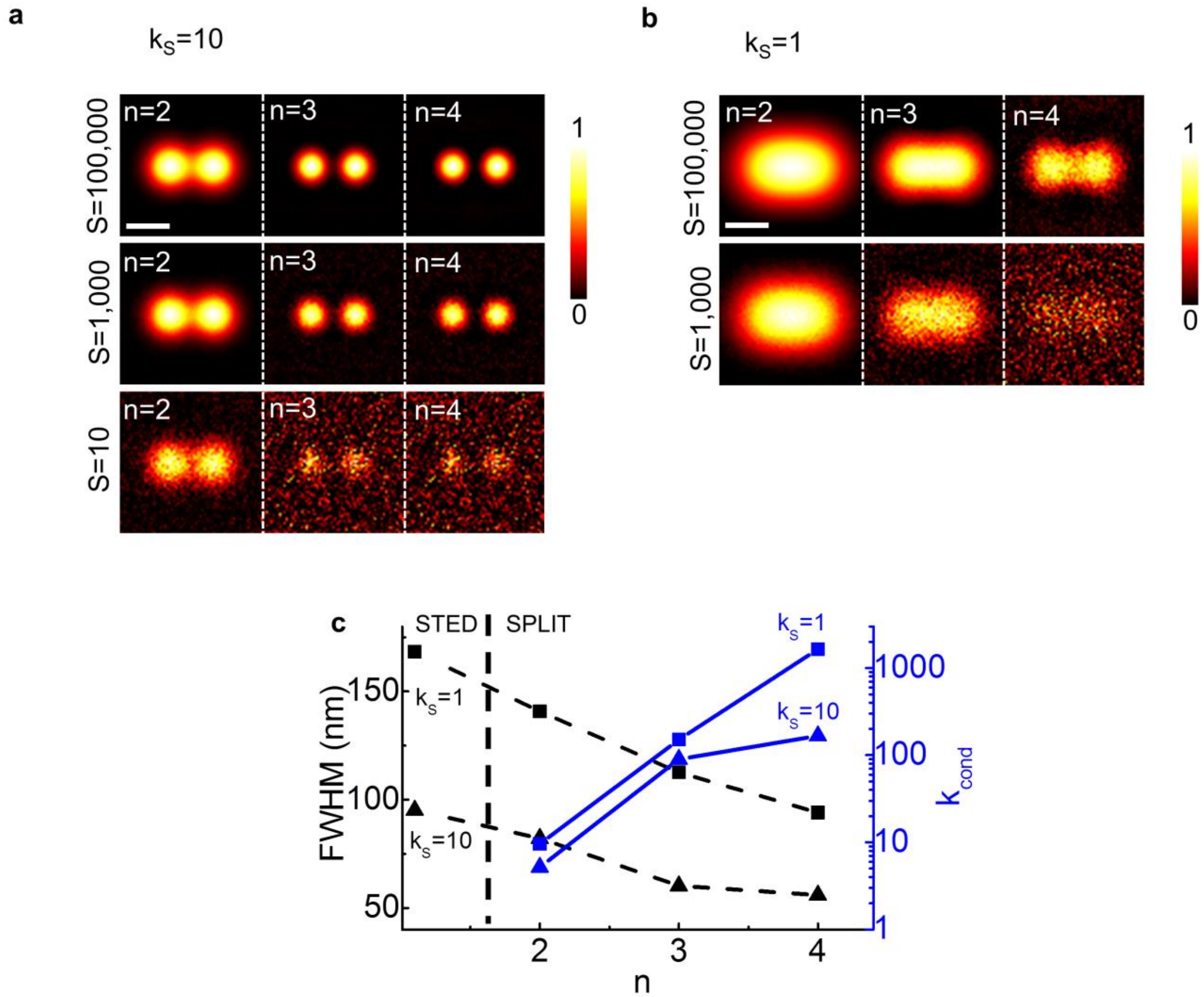

Supplementary Figure 4. SPLIT of simulated data in presence of noise (**a**,**b**) Simulations of SPLIT images (only first component is shown) obtained with different number of components *n* and in presence of noise. The parameters were set as follows: confocal FWHM=200 nm, particles distance = 104 nm, τ_0 = 2.5 ns. The noise was varied by changing the value of *S* as indicated in the figure. The signal-to-background ratio was kept to the constant value *S*/*B*=10 by adding a uniform level of background *B*. The SPLIT method was applied to time-resolved STED images with (**a**) k_S = 10 and (**b**) k_S = 1. The colormap represents the simulated intensity normalized to the maximum value of each image. Scale bars 100 nm. (**c**) Plot showing the dependence of the condition number k_{cond} of the matrix **M** as a function of the number of components *n* along with the variation of FWHM.

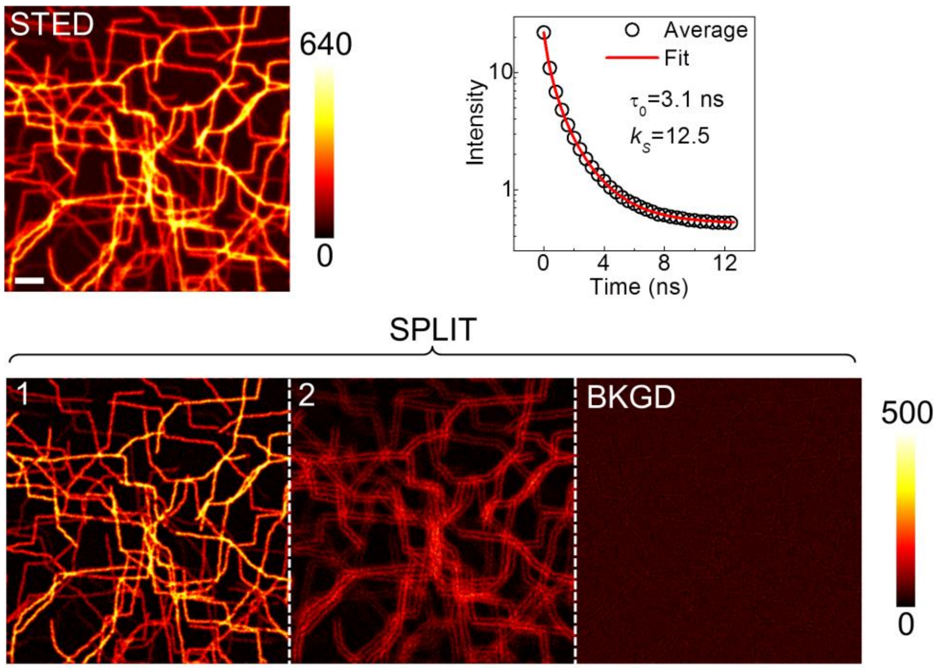

Supplementary Figure 5. **SPLIT of simulated STED imaging of cytoskeletal phantoms.** Simulated time-resolved STED image of fiber-like structures similar to those found in cytoskeletal networks. The input parameters were: τ_0 = 3.1 ns, k_S = 12.7, S = 120, B = 0.5. The average STED decay is fitted to equation (3) to obtain the parameter k_S (k_S = 12.5 from the fit in keeping with the input). The parameters τ_0 and k_S are then used to split the STED image in the SPLIT series (n=2) (component 1, component 2, background (BKGD)). The colormap represents the simulated intensity. Scale bar 1 μm.

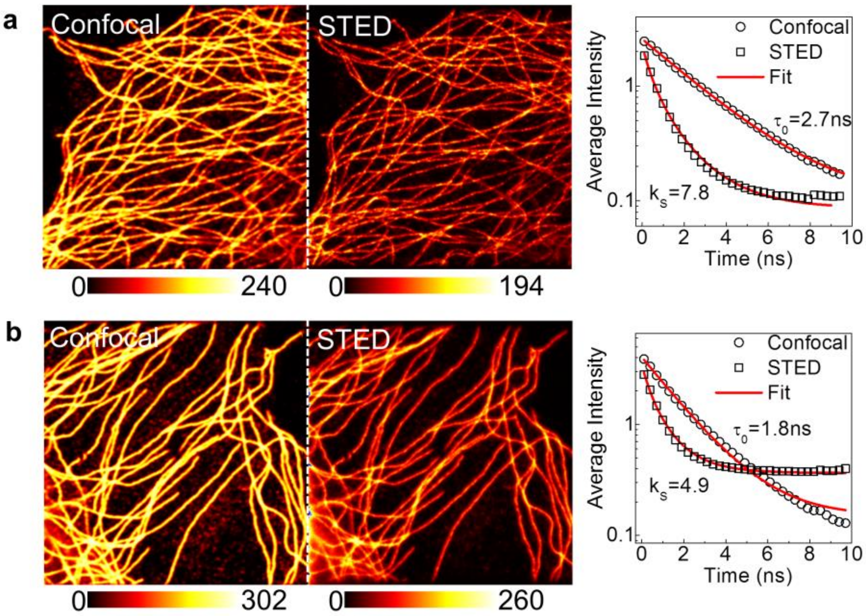

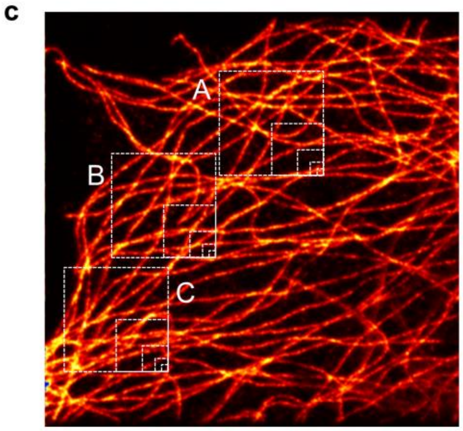

ROI Size (pixels)	A	B	C
128	7.8	7.9	8
64	7.9	8	8.2
32	8.0	8.4	7.8
16	8.0	8.7	8.6
8	8.1	5.3	7.4
4	9.1	7.6	5.5

Supplementary Figure 6. Determination of decoding parameters from experimental biological imaging data. Time-resolved confocal and STED images of microtubules in fixed HeLa cells labeled by immunocytochemistry with the organic dyes Alexa Fluor 488 (**a**), and Oregon-Green (**b**) (image size = 20 μm × 20 μm). Shown are the confocal image, the STED image, the average decay from all the pixels of both confocal and STED along with the fit according to equation (3) in main text. For the confocal decay we fix $k_S = 0$. For the STED we fix τ_0 to the value obtained from the confocal fit. The STED power measured at the back aperture of the objective lens was P_{STED} = 40mW. (**c**) Extraction of the parameter k_S in smaller regions of interest (ROI) of different size selected from (**a**). The values of k_S are reported in the corresponding table.

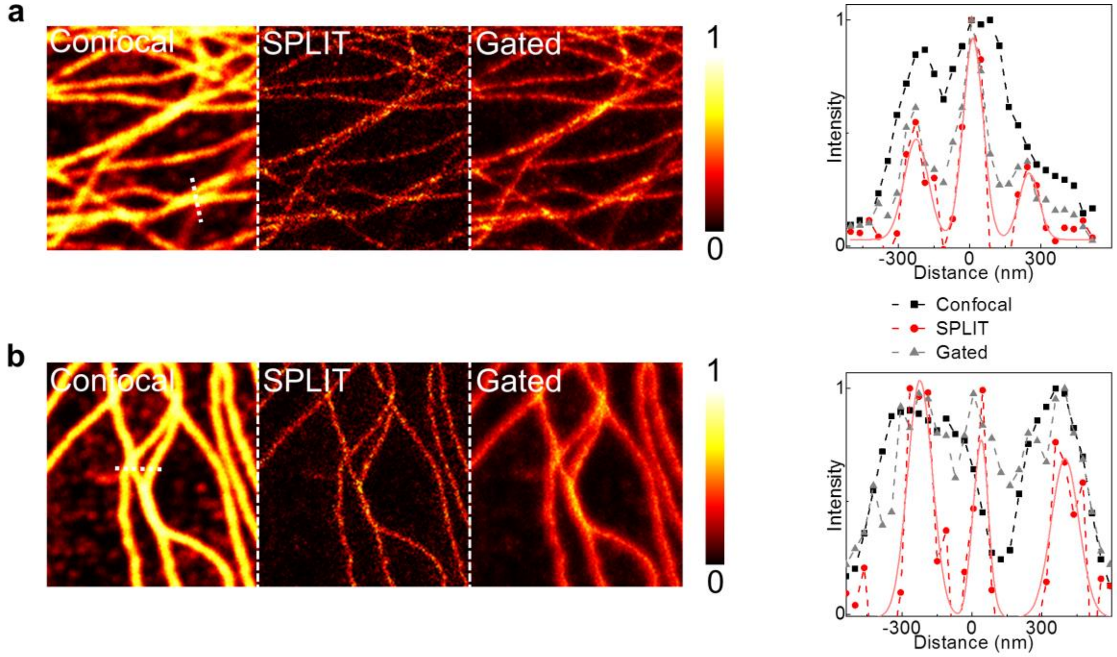

Supplementary Figure 7. Alternative regions of interest for the biological imaging data. Regions of interest (image size=5.5 μm × 5.8 μm) extracted from the full field of view showing microtubules in fixed HeLa cells labeled by immunocytochemistry with the organic dyes Alexa Fluor 488 (**a**), and Oregon Green 488 (**b**). Shown are the confocal image, the SPLIT (*n*=2, first component) image, the time-gated image (T_g=1 ns) and the intensity profile along the dashed line. The colormap represents the fluorescence intensity normalized to the maximum value of each image.

Supplementary Notes

Supplementary Note 1 - Mathematical modeling of stimulated emission induced lifetime variations

Modeling of the STED decay components

For simplicity of calculation, we assume a 3D Gaussian profile of the confocal PSF, with waists along the x, y and z directions given by w_x=w_y=w and w_z respectively:

$$h(x',y',z') = \exp\left(-2\left(x'^2+y'^2\right)/w^2\right)\cdot\exp\left(-2z'^2/w_z{}^2\right) = h_r\left(r^2\right)h_z(z'),$$

where we have defined the radial part of the confocal PSF as:

$$h_r\left(r^2\right) = \exp\left(-2r^2/w^2\right),$$

with $r^2 = x'^2 + y'^2$.

Then we approximate the doughnut-shaped intensity distribution of the STED beam at the focus as a parabolic function of the radius and ignore for simplicity any dependence along z:

$$I_{STED}(r) \approx I_{STED}(w)\frac{r^2}{w^2}.$$

The instantaneous probability of stimulated emission depends linearly on the STED beam intensity:

$$\gamma_{STED}\left(r^2\right) = \gamma_0\left[I_{STED}(r)/I_{SAT}\right],$$

where $\gamma_0=1/\tau_0$ is the decay rate of the spontaneous emission and the constant I_{SAT} is usually called saturation intensity[1] and represents the value of intensity for which $\gamma_{STED}=\gamma_0$.

The resulting decay rate as a function of the position is:

$$\gamma(r^2)=\gamma_0+\gamma_{STED}(r^2)=\gamma_0+\gamma_0[I_{STED}(r)/I_{SAT}]=\gamma_0+\gamma_0[I_{STED}(w)(r^2/w^2)/I_{SAT}]=\gamma_0+\gamma_0 k_S r^2/w^2 .$$

where we have defined:

$$k_S=I_{STED}(w)/I_{SAT} .$$

The time-dependent fluorescence intensity $F(x,y,t)$ at each pixel can be expressed as:

$$F(x,y,t)=K\int e^{-\gamma(r^2)t}\rho(x',y',z')e^{-\frac{2r^2}{w^2}-\frac{2z'^2}{w_z^2}}dx'dy'dz',$$

where K is a constant that depends on the quantum yield of the fluorophore, the maximum of the excitation intensity and the detection efficiency, $r^2=(x'-x)^2+(y'-y)^2$ and $\rho(x',y',z')$ is the density of fluorophores. We conveniently switch to a system of cylindrical coordinates centered on the pixel (x,y) and integrate along z' and ϕ':

$$F(x,y,t)=K\int_0^\infty e^{-\gamma(r^2)t}e^{-\frac{2r^2}{w^2}}\int_0^{2\pi}\int_{-\infty}^{\infty}\rho(r,\phi',z')e^{-\frac{2(z'-z)^2}{w_z^2}}rdrd\phi'dz'=K\int_0^\infty C(r^2)dr^2e^{-\gamma(r^2)t}e^{-\frac{2r^2}{w^2}},$$

with $C(r^2)dr^2=\int_0^{2\pi}\int_{-\infty}^{\infty}\rho(r',\phi',z')e^{-\frac{2(z-z')^2}{w_z^2}}rdrd\phi'dz'$. With this definition, $C(r)$ describes the effective concentration of fluorophores in a concentric cylinder of radius r around the pixel position and z profile described by $h_z(z')$.

Discretization of the continuous distribution of STED decay components

In order to approximate the continuous distribution of decays in a discrete number n of components, we split the integral into n parts:

$$F(x,y,t) = K\int_0^{r_1} C(r^2)dr^2 e^{-\gamma(r^2)t} e^{-\frac{2r^2}{w^2}} + \ldots + K\int_{r_{n-1}}^{\infty} C(r^2)dr^2 e^{-\gamma(r^2)t} e^{-\frac{2r^2}{w^2}}.$$

We need to get n components which do not depend on the function $C(r^2)$. For this reason we expand $C(r^2)$ as a Fourier series inside the interval (r_{i-1}^2, r_i^2) of width Δr_i^2:

$$C(r^2) = C_0^i + C_1^i \sin\left[2\pi\left(r^2 - r_{i-1}^2\right)/\Delta r_i^2 + \varphi_i\right] + \ldots$$

and approximate the function $C(r^2)$ with the term of order zero:

$$C(r^2) \approx C_0^i.$$

Then we can write:

$$\int_{r_{i-1}}^{r_i} C(r^2)dr^2 e^{-\gamma(r^2)t} e^{-\frac{2r^2}{w^2}} \approx C_0^i \int_{r_{i-1}}^{r_i} dr^2 e^{-\gamma(r^2)t} e^{-\frac{2r^2}{w^2}}.$$

We are approximating $C(r^2)$ inside (r_{i-1}^2, r_i^2) with its average value within this interval, and ignoring its variations inside (r_{i-1}^2, r_i^2).

We define the time-dependent decay of the ith component as:

$$I_i(t) = \int_{r_{i-1}}^{r_i} dr^2 e^{-\gamma(r^2)t} e^{-\frac{2r^2}{w^2}} \propto e^{-\gamma_0 t} \frac{1}{1+k_S\gamma_0 t/2}\left(e^{-(1+k_S\gamma_0 t/2)2r_{i-1}^2/w^2} - e^{-(1+k_S\gamma_0 t/2)2r_i^2/w^2}\right).$$

Then we can write the intensity as a linear combination of components:

$$F(x,y,t) \approx K \sum_{i=1}^{n} C_0^i(x,y) I_i(t).$$

The boundaries r_i of the subdiffraction volumes (besides the extreme values $r_0 = 0$ and $r_n = \infty$) are chosen in such a way that:

$$\int_0^T I_i(t)dt = \frac{1}{n} \sum_{i=1}^{n} \int_0^T I_i(t)dt \qquad \text{for } i = 1...n.$$

With this choice, in the particular case of $C(r^2)$ = constant (homogeneous distribution of fluorophores), all the time-correlated photons, N-N_{BKGD} , are split in equal number among the n components.

A smaller value of the ratio r_1/w is associated to a resolution improvement of the first image of the SPLIT series with respect to the confocal image. The FWHM of the effective PSF of the SPLIT image decreases with increasing values of n as shown in Supplementary Fig. 2 for the values $k_S = 10$, $k_S = 1$ and $k_S = 0.1$.

If we substitute the function $C(r^2)$ with a constant C, the analytical form of the decay is given by:

$$F(x,y,t) = KC \int_0^\infty dr^2 e^{-\gamma(r^2)t} e^{-\frac{2r^2}{w^2}} \propto e^{-\gamma_0 t} \frac{1}{1 + k_S \gamma_0 t/2}.$$

This function can be a good approximation for the average time-resolved decay of all the pixels of an image. Indeed, consider the simple case of an image obtained scanning around a point-like object, for which $C(r^2) \sim \delta(r^2)$:

$$\iint_{x,y} F(x,y,t)dxdy = K\iint_{x,y} dxdy\int_0^\infty C(r^2)dr^2 e^{-\gamma(r^2)t} e^{-\frac{2r^2}{w^2}} \approx K\int_0^\infty dr^2 e^{-\gamma(r^2)t} e^{-\frac{2r^2}{w^2}} \propto e^{-\gamma_0 t}\frac{1}{1+k_S\gamma_0 t/2}.$$

If we take into account the presence of uncorrelated background we obtain the following functional form:

$$\iint_{x,y} F(x,y,t)dxdy = Ae^{-\gamma_0 t}\frac{1}{1+k_S\gamma_0 t/2} + B,$$

which can be used to extract the parameter k_S from the experimental data.

Supplementary Note 2 – Explicit expression of the SPLIT image

The first component of the SPLIT image (n=2) can be explicitly expressed as a function of the phasor components:

$$N_1(x,y) = f_1(x,y)N(x,y) = \left[M_{11}^{-1}g(x,y) + M_{12}^{-1}s(x,y)\right]N(x,y)$$

Where $N(x,y)$ is the time-integrated intensity and $\mathbf{M}^{-1}$ is the inverse of the matrix **M**. The images of the phasor components $g(x,y)$ and $s(x,y)$, extracted from the temporal dynamics, potentially encode the additional spatial information. This extra amount of information is evident for instance in the case of a CW-STED image whereas is absent in a confocal image (see Supplementary Fig. 3 where the images of $\tau_{phase}(x,y)=(T/2\pi)(s(x,y)/g(x,y))$, a parameter independent from the amount of background, are reported).

We can explicitly express the above equation as a function of the time-resolved fluorescence intensity and obtain:

$$N_1(x,y) = M_{11}^{-1}\int_0^T F(x,y,t)\cos(2\pi t/T)dt + M_{12}^{-1}\int_0^T F(x,y,t)\sin(2\pi t/T)dt$$

Now consider the case of CW-STED, where the temporal evolution of $F(x,y,t)$ encodes spatial information through the spatial dependence of $\gamma(r^2)$. By inserting the expression for $F(x,y,t)$ we obtain:

$$N_1(x,y) = K\int\left[M_{11}^{-1}\int_0^T e^{-\gamma(r^2)t}\cos(2\pi t/T)dt + M_{12}^{-1}\int_0^T e^{-\gamma(r^2)t}\sin(2\pi t/T)dt\right]\rho(x',y',z')e^{-\frac{2r^2}{w^2}-\frac{2z'^2}{w_z^2}}dx'dy'dz'$$

Which describes analytically the image process formation of the SPLIT technique.

Notably, the SPLIT imaging technique is still a linear and space-invariant system. The later properties vanish if the parameter k_S changes across the sample. Similarly, a STED microscope is a space-invariant system only if k_S is constant across the sample. For this reason it could be useful to depict the effective PSF for the SPLIT approach as:

$$EPSF_{SPLIT}^{n=2}(x,y) = \left[M_{11}^{-1}\int_0^T e^{-\gamma(r^2)t}\cos(2\pi t/T)dt + M_{12}^{-1}\int_0^T e^{-\gamma(r^2)t}\sin(2\pi t/T)dt\right]e^{-\frac{2r^2}{w^2}-\frac{2z'^2}{w_z^2}},$$

and the image process formation as a convolution between the effective PSF and the fluorophore concentration ρ. The first part of the *E-PSF* expression clearly shows the fusion of the spatio-temporal information through the function $\gamma(r^2)$.

Depending on the coefficients M_{ij}^{-1} which are obtained from the inversion of the decoding matrix **M**, this formula acts as a spatial filter by selecting the pixels characterized by a temporal dynamics similar to the first dynamics component. It should be noted that, in the specific case of

CW-STED, for low values of k_S the separation into only n=2 components may not lead to significant reduction of the SPLIT *E-PSF*. If this is the case, further improvement in resolution can be obtained by separation into a larger number n of components, corresponding to an effective $E\text{-}PSF^{n}_{SPLIT}$ containing additional terms which extract spatial information from higher harmonic content of the temporal dynamics.

Supplementary Note 3 - Propagation of the noise in the SPLIT method

The SPLIT image, $N_i(x,y)=f_i(x,y)N(x,y)$, is affected by additional noise brought in by the factor $f_i(x,y)$. The fraction f_i is calculated at each pixel from the measurement of g and s at one or multiple harmonics. Here we discuss how the noise in the measurement of g and s (or higher harmonics components) is propagated to the fractions f_i in the linear system $\mathbf{P}=\mathbf{Mf}$ depending on the properties of the matrix **M**. The error propagation through a linear system is usually quantified by considering the 'condition number' k_{cond} defined as the product of the norms of the matrices **M** and its inverse $\mathbf{M}^{-1}$:

$$k_{cond}=\|\mathbf{M}\|\|\mathbf{M}^{-1}\|$$

The parameter k_{cond} is dependent on the particular choice of the n vectors (g_1, s_1,…), … , (g_n, s_n,…) which form the matrix **M** and which describe the n dynamics components. This parameter decreases with higher values of k_S and increases with the value of n. This dependence is shown for the values k_S = 1 and k_S = 10 in Supplementary Fig. 4. It can be seen from the figure that it is possible, in principle, to get with the value k_S =1 (n = 4) a resolution comparable to that obtained with k_S = 10 (n=2) but with a noise propagation which is 2 orders of magnitude larger. Because

of the exponential dependence of k_{cond} with respect to n, in most of the practical cases, noise may limit the use of SPLIT for high values of n.

Supplementary References

1. Vicidomini, G. et al. Sharper low-power STED nanoscopy by time gating. *Nat. Methods* **8**, 571-573 (2011).
